# Apical longitudinal strain: A Key prognostic echocardiographic marker in patients undergoing transcatheter aortic valve implantation

**DOI:** 10.1016/j.ijcha.2025.101844

**Published:** 2025-11-19

**Authors:** Yuichiro Shirahama, Hiroki Usuku, Eiichiro Yamamoto, Tatsuya Yoshinouchi, Ryudai Higashi, Atsushi Nozuhara, Fumi Oike, Noriaki Tabata, Masanobu Ishii, Shinsuke Hanatani, Tadashi Hoshiyama, Hisanori Kanazawa, Yuichiro Arima, Hiroaki Kawano, Yasuhiro Izumiya, Yasuhito Tanaka, Kenichi Tsujita

**Affiliations:** a Department of Cardiovascular Medicine, Graduate School of Medical Sciences, Kumamoto University, Kumamoto, Japan; b Center of Metabolic Regulation of Healthy Aging, Kumamoto University Faculty of Life Sciences, Kumamoto, Japan; c Department of Laboratory Medicine, Kumamoto University Hospital, Kumamoto, Japan; d Department of Gastroenterogy, Kumamoto University Hospital, Kumamoto, Japan

**Keywords:** Apical longitudinal strain of the left ventricle, 2D speckle tracking echocardiography, Transcatheter aortic valve implantation

## Abstract

**Background:**

Although the association between global longitudinal strain (GLS), a marker of myocardial systolic function, and prognosis in patients undergoing transcatheter aortic valve implantation (TAVI) is well-documented, the prognostic association of regional longitudinal strain (LS), such as apical LS, on patients undergoing TAVI remains underexplored.

**Methods and Results:**

From 2015 to 2023, a total of 303 patients with aortic stenosis (AS) who underwent TAVI at Kumamoto University Hospital were screened, and excluding 4 patients with in-hospital deaths, 299 were analyzed. The median follow-up period after TAVI was 693 days (interquartile range, 435–1189 days), during which 63 deaths occurred. Pre-TAVI echocardiographic findings showed that apical LS was significantly higher in the survival group compared to the all-cause death group (15.1 ± 4.7％ vs. 13.7 ± 4.4 %, p = 0.02). Multivariable Cox proportional hazards analysis, adjusted for body mass index, aortic valve peak velocity, atrial fibrillation, high-sensitivity troponin T, tricuspid regurgitation, demonstrated that apical LS was independently associated with all-cause mortality (hazard ratio: 0.91, 95 % confidence interval: 0.88–0.99, p = 0.02). Time-dependent receiver operating characteristic (ROC) curve analysis identified apical LS to discriminate all-cause mortality (area under the curve, 0.69), with the predictive ability peaking within the first two years after TAVI. Kaplan–Meier analysis revealed significantly higher mortality rates in patients with low apical LS group (<15.4 %) (p = 0.01).

**Conclusions:**

measurement of apical LS in patients with AS provides valuable associational prognostic information, even after adjusting for multiple clinical and echocardiographic factors, highlighting its value in enhancing risk stratification for patients undergoing TAVI.

## 1 Introduction

Transcatheter aortic valve implantation (TAVI) has revolutionised the treatment of severe aortic stenosis (AS) and offers a less-invasive alternative to surgical valve replacement in high-risk patients [1]. To evaluate the prognosis of the patients who underwent TAVI, conventional echocardiographic parameters (ex. Reduced left [2] or right [3] ventricular function and the severity of AS [4]) have proven to be useful. However, they have inherent limitations in detecting subtle myocardial dysfunctions.

In this context, 2D speckle-tracking echocardiography has emerged as a powerful tool that provides a more detailed assessment of myocardial deformation [[5], [6], [7]]. Global longitudinal strain (GLS) has been established as a robust marker of left ventricular(LV) systolic function and an independent predictor of clinical outcomes in various cardiac disease, including TAVI [8,9]. However, previous reports on the utility of GLS in TAVI are limited in number and heterogeneous in methodology, making its prognostic utility still uncertain. Moreover, GLS represents an averaged value across the entire ventricle and may overlook region-specific differences in myocardial function.

Recent studies have highlighted the importance of the regional components of longitudinal strain in various cardiac conditions, such as the LV apical-sparing pattern observed in cardiac amyloidosis, which underscores the diagnostic and prognostic potential of regional strain analysis [10,11]. However, GLS does not allow the evaluation of individual regional longitudinal strain(LS) components, such as apical, mid, and basal LS. The prognostic value of the regional components remains unclear in the context of TAVI. The LV apex, often less affected by concentric remodelling in AS, may serve as a sensitive indicator of residual cardiac dysfunction through apical longitudinal strain (apical LS) [12].

This study aimed to fill this knowledge gap by investigating the relationship between apical LS and all-cause mortality in patients who underwent TAVI. By employing comprehensive echocardiographic evaluations and survival analyses, we aimed to elucidate the prognostic significance of apical LS and its potential role in enhancing the risk stratification of this high-risk population.

## 2 Methods

### 2.1 Study population

This study included 303 patients with AS who underwent TAVI at the Kumamoto University Hospital between June 2015 and February 2023. Four patients who died during the index hospitalization were excluded because their echocardiographic parameters may not have represented stable baseline cardiac function prior to the procedure. These deaths were mainly related to procedural or acute postoperative complications rather than underlying myocardial dysfunction. Consequently, 299 patients were included in the analysis. Baseline clinical characteristics, laboratory results, and echocardiographic findings were collected when the patients were in a clinically stable condition prior to the TAVI procedure. Clinically stable condition was defined as the absence of cardiogenic shock, no use of inotropes or vasopressors, systolic blood pressure ≥ 90 mmHg, and hemodynamic stability without mechanical circulatory support. In this study, all patients underwent elective TAVI and none presented with NYHA class IV symptoms at rest, cardiogenic shock, or required urgent/emergency procedures. At our institution, patients with ≥ 90 % diameter stenosis or functionally significant coronary artery disease undergo PCI prior to TAVI. This retrospective study adhered to the principles of the Declaration of Helsinki and was approved by the institutional review board of Kumamoto University (No. 1588). The requirement for informed consent was waived due to the minimal risk of patient identification. The study details were made publicly available at the Kumamoto University Hospital and on the institution’s website to provide patients with the opportunity to opt out of the study.

### 2.2 Conventional echocardiographic measurements

Pre-TAVI transthoracic echocardiography was performed under stable clinical conditions using various ultrasound systems (Vivid E95 or 7, Aplio 500, and Epiq 7G) with a 2.5-MHz transducer. Imaging was performed according to the American Society of Echocardiography (ASE) and European Association of Cardiovascular Imaging guidelines [13]. Frame rates were maintained between 50 and 90 frames per second [14], and sector width was minimized to optimize temporal resolution. Vendor-specific presets were harmonized to ensure consistency across different scanners. Left ventricular (LV) wall thickness was measured in the parasternal long-axis view, and LV ejection fraction (LVEF) was calculated using the modified Simpson’s method. Additional parameters such as E velocity and e′ velocity were assessed from the apical four-chamber view. Moderate or severe valvular disease was classified based on ASE guidelines [15]. In patients with frequent ectopic beats or paced rhythm, as well as in those with atrial fibrillation, three cardiac cycles with stable R–R intervals were averaged for strain analysis. Cases with more than moderate valvular regurgitation were included in the analysis. Heart rate was recorded at the time of image acquisition, although blood pressure was not routinely measured. All images were obtained when patients were hemodynamically stable without requiring inotropic or vasopressor support. To prevent bias, echocardiographic reviewers were blinded to the patients’ clinical histories and data.

### 2.3 Two-Dimensional Speckle-Tracking echocardiography

Two-dimensional speckle-tracking echocardiography was performed using cardiac performance analysis with a manual vendor-independent measurement package (TomTec-Arena version 4.6; TomTec Imaging Systems, Unterschleissheim, Germany) and the same algorithm was applied throughout the study. The LV longitudinal strain (LS) was derived from four-, three-, and two-chamber views and averaged over 16 LV segments according to the ASE guidelines [13]. Additionally, the LV longitudinal strain was divided into three regional components: apical longitudinal strain (apical LS), mid-ventricular longitudinal strain (mid LS), and basal longitudinal strain (basal LS). These regional LS values were analysed separately to assess their respective predictive values for clinical outcomes. Basal, mid, and apical LS were defined according to the standard 16-segment model recommended by the ASE: basal segments (1–6), mid segments (7–12), and apical segments (13–16). Poor-tracking segments were excluded a priori. Studies with more than two non-analyzable segments would have been excluded from the analysis; however, no case met this exclusion criterion. Strain values were expressed as negative numbers, with more negative values indicating better LV systolic function. Absolute strain values were used for statistical analyses.

Both conventional echocardiography and strain analysis were performed by experienced observers who were blinded to all clinical and outcome data. Conventional echocardiography was reviewed by experienced cardiologists, and strain analysis was performed by an independent operator. For reproducibility testing, two independent observers re-analyzed a randomly selected subset of cases without access to any clinical or outcome information. All analyses were performed offline to ensure complete blinding. To evaluate measurement reliability, interobserver and intraobserver reproducibility were assessed. Two independent observers measured LS in 21 randomly selected cases. Interobserver and intraobserver reproducibility were evaluated using the intraclass correlation coefficient (ICC) based on a two-way random-effects model with absolute agreement and average-measure definition. 95 % CI were calculated using the F distribution.The ICC for interobserver reliability of apical LS was 0.858 (95 % CI: 0.657–0.942), and the ICC for intraobserver reliability was 0.965 (95 % CI: 0.911–0.986), indicating excellent reproducibility.

### 2.4 Data Collection

Mortality data were obtained from hospital records and confirmed via questionnaires or telephone interviews with patients or their families, with follow-up finalised in September 2024.

### 2.5 Statistical analysis

Continuous variables were expressed as means ± standard deviation, while non-normally distributed data (e.g. high-sensitivity cardiac troponin T (hs-cTnT) and B-type natriuretic peptide (BNP) levels) were log-transformed. Group comparisons were performed using Student’s *t*-test or chi-squared test. Cox proportional hazards regression analyses were conducted to identify the predictors of all-cause mortality. Two multivariable Cox proportional hazards models were pre-specified: one including apical LS and the other including GLS as the main exposure variable, along with relevant clinical covariates. This approach was chosen to avoid multicollinearity between apical LS and GLS, which are derived from overlapping myocardial segments. The proportional hazards assumption for the Cox regression model was verified using Schoenfeld residuals, and no statistically significant violation was observed for any covariate or for the global test. All data used for the multivariable analyses were complete, and no imputation procedures were required. Variables with a univariate p-value < 0.05 and clinical significance were included in the multivariable model. Time-dependent Receiver operating characteristic (ROC) curves and the area under the curve (AUC) were calculated to evaluate the discriminatory performance of apical LS. Kaplan–Meier analyses were used to compare survival outcomes. Patients were dichotomized at the cohort median apical LS. Statistical analysis was conducted using SPSS version 26.0, with p < 0.05 indicating statistical significance [16].

## 3 Results

### 3.1 Comparison of clinical characteristics between All-Cause death and survival groups

The median interval between preprocedural echocardiography and the TAVI procedure was 37 days (IQR: 9–58) for the overall cohort, 39 days (IQR: 17.5–64) for the survival group, and 26 days (IQR: 2–56) for the non-survival group. There were no clinically significant hemodynamic or clinical status changes during this period in any patient. A median follow-up period of 693 days (interquartile range, 435–1189 days) revealed 63 deaths among the cohort. Follow-up completeness was 94 % at 1 year, 85 % at 2 years, 77 % at 3 years, 63 % at 4 years, and 36 % at 5 years. The causes of death included heart failure (n = 8); sudden death outside the hospital (n = 3); cerebrovascular events such as infarction or haemorrhage (n = 6); pneumonia (n = 13); sepsis (n = 6); cancer (n = 7); general weakness (n = 6); renal failure (n = 2); and unknown causes (n = 11). Pre-procedural PCI was performed in 19/63 (30.2 %) patients in the death group and 57/236 (24.2 %) in the survival group (p = 0.41). Table 1 outlines the clinical characteristics of patients in the all-cause and survivor groups.

**Table 1:** Baseline clinical characteristics, laboratory findings, echocardiographic findings and treatments between all-cause death group and survival group.

	**All-cause Death Group (n = 63)**	**Survival Group (n = 236)**	**p-value**
**Baseline characteristics**			
Age at diagnosis, years	86.2 ± 5.8	85.1 ± 4.8	0.12
Male sex, n (%)	27 (40)	78 (33)	0.24
Body mass index, kg/m^2^	21.4 ± 3.6	22.6 ± 3.5	0.01
Past medical history			
Hypertension, n (%)	51 (76)	184 (78)	0.74
Diabetes mellitus, n (%)	21 (31)	55 (23)	0.2
Dyslipidemia, n (%)	33 (49)	133 (56)	0.33
Smoking, n (%)	4 (6)	7 (3)	0.29
Previous MI, n (%)	4 (6)	6 (3)	0.24
Previous PCI, n (%)	19 (30)	57 (24)	0.41
Atrial fibrillation, n (%)	12 (18)	23 (11)	0.14
STS-PROM, % (median [IQR])	4.8 (3–7)	3.7 (3–6)	0.03
Low risk (<4%), n (%)	25 (40)	122 (52)	0.11
Intermediate risk (4–8 %), n (%)	31 (49)	97 (41)	0.24
High risk (≥8%), n (%)	7 (11)	17 (7)	0.43
**Methods of TAVI**			
Transfemoral approach, n (%)	61 (91)	220 (93)	0.6
Balloon expandable valve, n (%)	40 (60)	172 (73)	0.04
Valve-in-valve, n (%)	1 (2)	7 (3)	0.2
**Laboratory findings before TAVI procedure**			
hs-cTnT, ng/mL	0.06 ± 0.19	0.03 ± 0.06	0.08
Log-transformed hs-cTnT	−3.46 ± 0.83	−3.72 ± 0.72	0.02
BNP, pg/ml	330 ± 350	268 ± 348	0.23
Log-transformed BNP	5.35 ± 0.94	5.08 ± 1.05	0.06
eGFR, ml/min/1.73 m^2^	44.0 ± 15.0	49.9 ± 16.8	0.02
**Echocardiographic findings before TAVI procedure**			
LAVI, ml/m^2^	56.5 ± 18.5	55.7 ± 18.8	0.83
IVSTd, mm	13.0 ± 2.1	12.5 ± 2.1	0.13
LVPWTd, mm	12.4 ± 2.2	12.0 ± 2.0	0.19
LVEF, %	60.0 ± 10.0	61.5 ± 8.6	0.22
E/e’ ratio	19.8 ± 7.2	18.4 ± 7.1	0.21
TRPG, mmHg	28.6 ± 9.2	27.1 ± 8.5	0.21
Aortic regurgitation, n (%)	2 (3)	15 (6)	0.38
Mitral regurgitation, n (%)	7 (10)	25 (10)	1
Tricuspid regurgitation, n (%)	12 (18)	20 (9)	0.04
LV-GLS, %	12.1 ± 3.1	12.8 ± 3.2	0.12
Apical LS	13.7 ± 4.4	15.1 ± 4.7	0.02
Mid LS	11.6 ± 3.3	11.7 ± 3.2	0.7
Basal LS	10.1 ± 3.5	10.6 ± 3.3	0.25
**Severity of aortic stenosis**			
Trans-aortic valve velocity, m/sec	4.45 ± 0.62	4.62 ± 0.68	0.07
Aortic valve area, cm^2^	0.60 ± 0.20	0.60 ± 0.18	0.83
**Treatments after TAVI procedure**			
RAS inhibitor, n (%)	40 (60)	111 (51)	0.26
CCB, n (%)	36 (54)	127 (59)	0.48
β-blocker, n (%)	21 (31)	52 (24)	0.26
MRA, n (%)	15 (22)	39(18)	0.47
SGLT-2 inhibitor, n (%)	3 (5)	4 (2)	0.36
Diuretics, n (%)	19 (45)	92 (39)	0.43

Abbreviations: MI, myocardial infarction; TAVI, transcatheter aortic valve implantation; hs-cTnT, high sensitivity cardiac troponin T; BNP, B-type natriuretic peptide; eGFR, estimated glomerular filtration rate; LAVI, left atrial volume index; IVSTd, interventricular septal thickness in diastole; LVPWTd, left ventricular posterior wall thickness in diastole; LVEF, left ventricular ejection fraction; TRPG, transtricuspid pressure gradient; LV-GLS, left ventricular-global longitudinal strain; RAS, renin angiotensin aldosterone system; CCB, calcium channel blocker; MRA, mineralocorticoid receptor antagonist; SGLT-2, sodium glucose cotransporter 2.

The p values were obtained by student’s *t*-test or chi-squared test.

There were no notable differences between the two groups in age at diagnosis, sex distribution, prevalence of hypertension, diabetes mellitus, dyslipidaemia, smoking, prior myocardial infarction (MI), or atrial fibrillation. However, body mass index (BMI) was significantly lower in the all-cause death group compared to the survival group (21.4 ± 3.6 kg/m^2^ vs. 22.6 ± 3.5 kg/m^2^, p = 0.01). The median STS-PROM score was 4.8 % (IQR 3.1–7.2) in the death group and 3.7 % (IQR 2.6–6.1) in the survival group. Regarding TAVI procedures, the use of balloon-expandable valves was less common in the all-cause death group compared to the survival group (60 % vs. 73 %, p = 0.04).

**Fig. 1:**
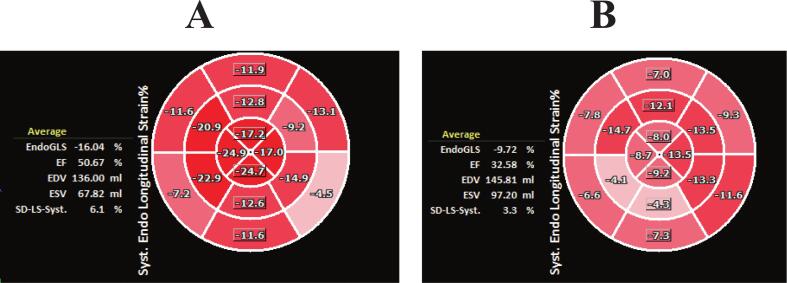
Typical findings of bull’s eye map in survival group(A), and in all-cause death group(B). A decrease in strain at the apex was observed in the all-cause death group.

Laboratory evaluations prior to TAVI revealed that log-transformed hs-cTnT levels were significantly higher in the all-cause death group (−3.46 ± 0.83 vs −3.72 ± 0.72, p = 0.02). However, there were no statistically significant differences in BNP levels between the two groups. The estimated glomerular filtration rate (eGFR) was significantly lower in the all-cause death group compared to the survival group (44.0 ± 15.0 ml/min/1.73 m^2^ vs 49.9 ± 16.8 ml/min/1.73 m^2^, p = 0.02). Echocardiographic assessments revealed a higher prevalence of tricuspid regurgitation (TR) in the all-cause death group than in the survival group (18 % vs. 9 %, p = 0.04). Furthermore, the apical longitudinal strain (apical LS) was significantly reduced in the all-cause death group compared to the survival group (13.7 ± 4.4 vs 15.1 ± 4.7, p = 0.02). The mean GLS and regional LS values in this cohort (GLS 12.5 ± 3.2 %, apical LS 14.4 ± 4.6 %, mid LS 11.6 ± 3.2 %, basal LS 10.3 ± 3.4 %) were comparable to those reported in previous TAVI studies [14,17], confirming that the present strain measurements were within physiologically expected ranges. Fig. 1 shows the typical findings of the bull’s eye map in the survival group(A) and all-cause death group(B). No statistically significant differences were observed in the severity of aortic stenosis or post-TAVI treatment between groups.

In the subgroup analysis comparing patients with high and low apical LS (Supplemental Table 1), the low apical LS group had a higher prevalence of atrial fibrillation, lower left ventricular ejection fraction, and a greater use of mineralocorticoid receptor antagonists (MRA). Other baseline clinical and procedural characteristics were comparable between the two groups. We also performed similar comparisons by GLS levels (data not shown) and confirmed consistent trends.

### 3.2 Cox proportional hazard analysis for All-Cause death

Univariate Cox proportional hazard analysis identified eight factors that were significantly associated with all-cause mortality: BMI, atrial fibrillation, log-transformed hs-cTnT level, eGFR, TR, transaortic valve velocity, global longitudinal strain (GLS), and apical LS (Table 2a). To address potential overlaps, multivariate analysis was conducted using two models: one excluding GLS (Model 1) and the other excluding apical LS (Model 2). The results indicated that apical LS remained an independently associated with all-cause death after adjusting for other factors, such as *trans*-aortic valve velocity, TR, atrial fibrillation, log-transformed hs-cTnT, eGFR, and BMI. Conversely, GLS was not independently associated with mortality in the adjusted models (Table2b).

**Table 2a:** Univariate Cox Proportional Hazards Model for All-cause Death in enrolled patients.

	Univariate analysis	
	HR (95 % CI)	P-value
Age/1 year increment	1.03 (0.98–1.09)	0.19
Male sex/ yes	1.48 (0.90–2.42)	0.12
Body mass index per 1 kg/m^2^	0.90 (0.84–0.98)	0.01
Hypertension/ yes	0.80 (0.45–1.39)	0.42
Diabetes mellitus/ yes	1.29 (0.77–2.16)	0.34
Dyslipidemia/ yes	0.63 (0.39–1.03)	0.06
Smoking/ yes	1.09 (0.39–2.96)	0.9
Previous MI/ yes	1.46 (0.53–4.02)	0.47
Atrial fibrillation/ yes	2.30 (1.22–4.32)	0.01
Transfemoral approach/ yes	0.63 (0.35–1.88)	0.81
Balloon expandable valve / yes	0.90 (0.54–1.50)	0.68
Log-transformed Hs-cTnT	1.43(1.09–1.88)	0.01
Log-transformed BNP	1.21(0.94–1.56)	0.13
eGFR/ 1 ml/min/1.73 m^2^	0.98 (0.97–1.00)	0.02
LAVI/1ml/m^2^	1.00 (0.98–1.02)	0.83
IVSTd/1mm	1.03 (0.89–1.20)	0.66
LVPWTd/1mm	1.02 (0.91–1.15)	0.74
LVEF/ 1 %	0.98 (0.96–1.01)	0.15
E/e’ ratio/1	1.01 (0.97–1.04)	0.83
TRPG/1mmHg	1.01 (0.98–1.03)	0.6
Aortic regurgitation/ yes	0.51 (0.12–2.06)	0.34
Mitral regurgitation/ yes	1.00 (0.46–2.20)	0.99
Tricuspid regurgitation/ yes	2.45 (1.30–4.60)	0.01
LV-GLS/ 1 %	0.92 (0.86–1.00)	0.02
Apical LS/ 1	0.93 (0.88–0.98)	0.002
Mid LS/1	0.98(0.91–1.06)	0.62
Basal LS/1	0.96(0.89–1.03)	0.24
Trans-aortic valve velocity/ 1 m/sec	0.63 (0.41–0.95)	0.03
Aortic valve area/ 1 cm^2^	1.02 (0.27–3.83)	0.98
RAS inhibitor/ yes	1.06 (0.65–1.73)	0.82
CCB/ yes	0.66 (0.41–1.07)	0.09
β-blocker/ yes	1.53 (0.91–2.57)	0.11
MRA/ yes	1.37 (0.77–2.44)	0.29
SGLT-2 inhibitor/ yes	5.6 (1.70–18.4)	0.05
Diuretics/ yes	1.55 (0.96–2.50)	0.07

Abbreviations: MI, myocardial infarction; hs-cTnT, high sensitivity cardiac troponin T; BNP, B-type natriuretic peptide; eGFR, estimated glomerular filtration rate; LAVI, left atrial volume index; IVSTd, interventricular septal thickness in diastole; LVPWTd, left ventricular posterior wall thickness in diastole; LVEF, left ventricular ejection fraction; TRPG, transtricuspid pressure gradient; LV-GLS, left ventricular-global longitudinal strain; RAS, renin angiotensin aldosterone system; CCB, calcium channel blocker; MRA, mineralocorticoid receptor antagonist; SGLT-2, sodium glucose cotransporter 2.

P value was obtained by the univariate Cox hazard analyses model.

**Table 2b:** Multivariable Cox Proportional Hazards Model for All-cause Death.

	Model 1		Model 2	
	HR (95 % CI)	p-value	HR (95 % CI)	p-value
Trans-aortic valve velocity/ 1 m/sec	0.67 (0.43 – 1.04)	0.08	0.66 (0.42 – 1.01)	0.06
moderate-severe TR	2.04 (1.01 – 4.14)	0.05	2.02 (0.99 – 4.09)	0.05
Atrial fibrillation/ yes	1.15 (0.55 – 2.40)	0.71	1.27 (0.61 – 2.64)	0.53
Log-transformed hs-cTnT/ 1	1.15 (0.83 – 1.57)	0.41	1.17 (0.84 – 1.62)	0.36
eGFR	0.99 (0.97 – 1.00)	0.11	0.99 (0.97 – 1.00)	0.11
Body mass index per 1 kg/m^2^	0.91 (0.84 – 0.99)	0.03	0.91 (0.84 – 0.99)	0.03
Apical LS	0.91 (0.88 – 0.99)	0.02		
LV-GLS/ 1 %			0.95 (0.87 – 1.04)	0.25

Abbreviations: hs-cTnT, high sensitivity cardiac troponin T; Apical LS, apical longitudinal strain; LV-GLS, left ventricular-global longitudinal strain; LA, left atrial.

p-value was obtained by the multivariate Cox hazard analysis.

### 3.3 Time-dependent ROC curve analysis for All-Cause death

**Fig. 2:**
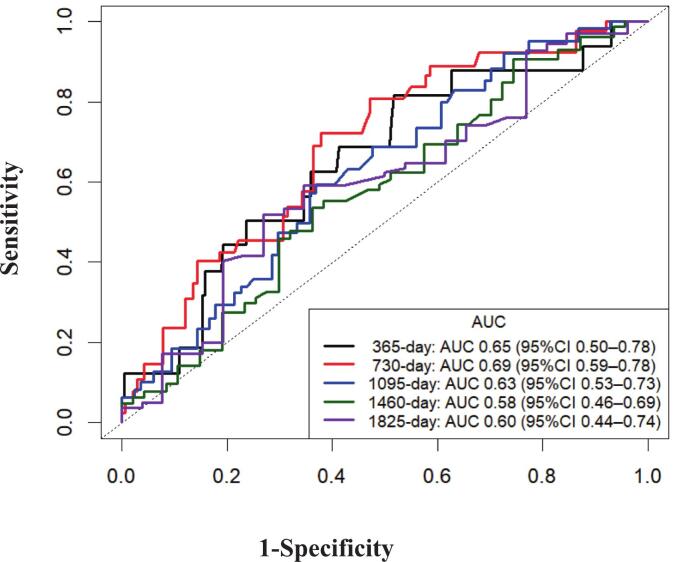
Time-dependent receiver operating characteristic curves of apical longitudinal strain for predicting all-cause mortality after transcatheter aortic valve implantation. The area under the curve was 0.65 (95 % CI: 0.50–0.78) at 365 days, 0.69 (95 % CI: 0.59–0.78) at 730 days, 0.63 (95 % CI: 0.53–0.73) at 1095 days, 0.58 (95 % CI: 0.46–0.69) at 1460 days, and 0.60 (95 % CI: 0.44–0.74) at 1825 days. The predictive ability of apical LS was highest within two years after TAVI. LS, longitudinal strain; AUC, area under the curve.

Time-dependent ROC curve analyses demonstrated that the AUC of apical LS for all-cause mortality was 0.65 (95 % CI: 0.50–0.78) at 1 year, 0.69 (95 % CI: 0.59–0.78) at 2 years, 0.63 (95 % CI: 0.53–0.73) at 3 years, 0.58 (95 % CI: 0.46–0.69) at 4 years, and 0.60 (95 % CI: 0.44–0.74) at 5 years (Fig. 2).

### 3.4 Follow-Up based on apical LS groups

**Fig. 3:**
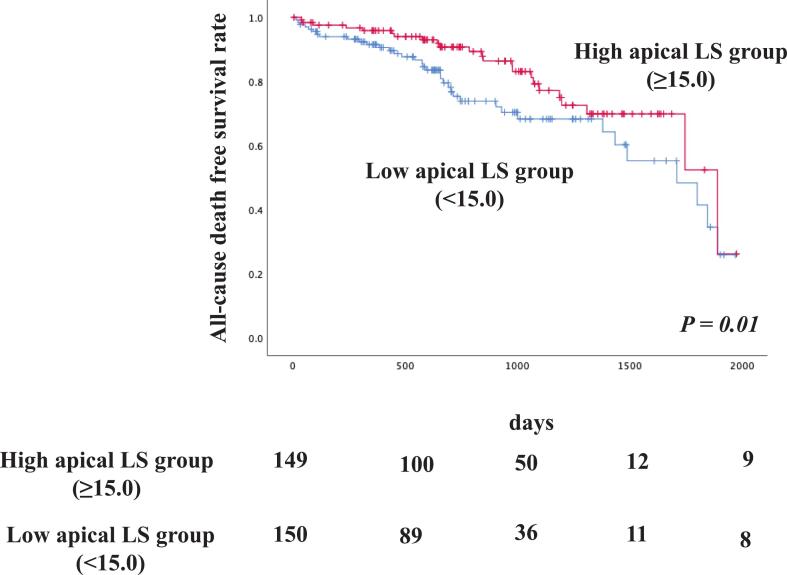
Kaplan–Meier curves for all-cause death in patients undergoing TAVI with high or low Apical LS. TAVI, Transcatheter aortic valve implantation; LS, longitudinal strain.

Patients were categorized into two groups according to the median apical LS value: high apical LS (≥15.0 %, n = 125) and low apical LS (<15.0 %, n = 153). Kaplan–Meier survival analysis revealed significantly higher all-cause mortality in patients with low apical LS than in those with high apical LS (p = 0.01, log-rank test; Fig. 3).

## 4 Discussion

In this study, we investigated the prognostic implications of echocardiographic parameters, including apical LS, in patients with severe AS who underwent TAVI. Our results demonstrate that reduced apical LS is independently associated with increased all-cause mortality, suggesting its potential as a prognostic indicator in this population. Conversely, GLS was not a significantly associated with mortality in multivariate analysis.

The finding that apical LS, rather than GLS, was more strongly associated with overall performance after TAVI. This extends previous evidence supporting the value of regional strain assessment in cardiac diseases and highlights apical LS as a simple and practical index for identifying high-risk patients.

The use of regional LS has also been reported [12]. Studies have demonstrated that when comparing pre- and post-TAVI parameters, mid-LS and basal LS showed statistically significant improvements compared to apical LS [18]. This finding is consistent with the understanding that pressure overload in AS predominantly affects the basal and midventricular segments, leaving the apex relatively spared [12,19]. This phenomenon, known as the apical-sparing pattern, reflects the localised myocardial function of the apex, which remains less influenced by pressure overload and fibrosis than other segments [20,21]. Several factors contribute to the relative preservation of apical function.

1) The apex experiences lower wall stress due to its reduced curvature and distinct myocardial fiber orientation, which distribute mechanical loads more efficiently [19,20].
2) Microcirculatory patterns and reduced fibrosis in the apex help maintain its contractile function, ensuring its continued contribution to overall cardiac output [[20], [21], [22]].
3) The unique architecture and fiber alignment of the apex may confer additional resilience against pathological remodeling, making it less susceptible to damage [23].

These combined factors likely result in the apical LS exhibiting higher values than in other regions, positioning it as a reliable indicator of residual cardiac function in AS. Consequently, the apical LS is a valuable metric for assessing myocardial health in this patient population.

Recent studies have highlighted the prognostic significance of the apical-sparing pattern in patients with AS undergoing TAVI. Ferreira et al. demonstrated better outcomes in patients with preserved apical LS, reinforcing its clinical relevance [12]. However, assessing apical-sparing patterns can be complex and time-consuming, limiting its feasibility in daily clinical practice. Our study builds on this finding by showing that apical LS alone is a simple yet effective prognostic marker. Time-dependent ROC analyses indicated a modest prognostic value of apical LS, which appeared most evident during the early follow-up period. However, given that AUCs were less than 0.70 and confidence intervals were wide, these results should be interpreted with caution. This suggests that apical LS primarily reflects early myocardial dysfunction relevant to mid-term outcomes, whereas long-term prognosis may be increasingly influenced by non-cardiac comorbidities and other clinical factors. Unlike the apical sparing pattern, which requires detailed and often labour-intensive measurements, apical LS provides a straightforward and practical alternative. By focusing on a single localised parameter, clinicians can streamline risk assessments without sacrificing accuracy, and ultimately improve patient care.

Moreover, the incorporation of apical LS into routine pre-TAVI evaluations could enhance risk stratification and guide individualised treatment strategies. This approach is aligned with the broader trend of integrating advanced echocardiographic techniques into clinical workflows to ensure that patients receive appropriate and effective care. As research continues to elucidate the mechanisms underlying’s prognostic value of the apical LS, its role in clinical practice is likely to expand, offering new opportunities for optimising outcomes after TAVI.

### 4.1 Limitations

This study has several limitations. First, as this was a single-centre retrospective study, caution is required when generalising the results. Although the majority of deaths in this study were classified as non-cardiovascular or of unknown cause, all-cause mortality was used as the primary endpoint because the number of adjudicated cardiovascular deaths was relatively small and some deaths labelled as “unknown” or due to “general weakness” might have been related to unrecognized cardiovascular events. Thus, we considered all-cause mortality to be a more robust endpoint in this elderly, frail TAVI population, but this choice may have diluted the specific association between strain parameters and strictly defined cardiovascular death. Second, the moderate sensitivity and specificity of the apical LS cut-off suggest that this indicator should not be used alone for assessing prognosis, but rather interpreted in conjunction with other clinical and imaging findings. Third, the follow-up period may not have been long enough to fully reflect long-term outcomes. Two-dimensional speckle-tracking echocardiography analysis was performed using vendor-independent software (TomTec Image-Arena). Although statistically significant correlations have been shown in the LS values analysed using vendor-independent software for paired images obtained from different ultrasound machines [24], this may introduce variability that should be considered when interpreting the results. Fourth, the prevalence of prior myocardial infarction and atrial fibrillation in our cohort was lower than that reported in large TAVI registries [25,26]. This likely reflects institutional selection and referral patterns, which may limit the generalizability of our findings to broader TAVI populations. Fifth, although several important clinical and echocardiographic parameters were adjusted for, potential residual confounding cannot be completely excluded. In particular, CT imaging parameters such as annular area, perimeter, and calcium score were not available in this study, which may have influenced the results. Finally, patients who experienced in-hospital mortality were excluded to minimize the impact of acute procedural complications on the analysis. However, this may have introduced potential selection or survivor bias, which should be considered when interpreting the findings.

### 4.2 Future Directions

Future research should focus on validating the prognostic value of Apical LS in larger multicentre studies with longer follow-up periods [27]. The incorporation of advanced imaging techniques such as cardiac MRI may also help to elucidate the relationship between myocardial fibrosis and changes in Apical LS in more detail [28,29]. Additionally, studies exploring the cellular and molecular mechanisms underlying apical sparing are of great interest [30]. Future studies should include serial echocardiographic evaluations to assess changes in GLS and regional LS after TAVI. Such analyses may provide valuable insights into regional recovery patterns and the mechanisms underlying the prognostic significance of apical LS.

## 5 Conclusion

Apical LS is associated with all-cause mortality in patients with AS who undergo TAVI, underscoring its potential as a prognostic tool in clinical practice. Integrating Apical LS into pre-TAVI evaluations may improve patient risk stratification and provide useful information for tailoring treatment strategies for individual patients.

## Data Availability Statement

The datasets used during the current study are available from the corresponding author on reasonable request.

## CRediT authorship contribution statement

**Yuichiro Shirahama:** Writing – original draft, Validation, Methodology, Investigation, Formal analysis, Data curation. **Hiroki Usuku:** Supervision, Investigation, Conceptualization. **Eiichiro Yamamoto:** Visualization, Validation, Supervision. **Tatsuya Yoshinouchi:** Methodology. **Ryudai Higashi:** Formal analysis, Data curation. **Atsushi Nozuhara:** Data curation. **Fumi Oike:** Formal analysis, Data curation. **Noriaki Tabata:** Supervision, Data curation. **Masanobu Ishii:** Visualization, Validation. **Shinsuke Hanatani:** Resources, Investigation. **Tadashi Hoshiyama:** Supervision, Methodology. **Hisanori Kanazawa:** Supervision. **Yuichiro Arima:** Visualization. **Hiroaki Kawano:** Visualization. **Yasuhiro Izumiya:** Investigation. **Yasuhito Tanaka:** Supervision. **Kenichi Tsujita:** Supervision.

## Funding

This study was supported in part by a Grant-in-Aid for Scientific Research (grant number 20 K08476) from the Japan Society for the Promotion of Science and a research grant from Edwards Lifesciences to HU.

## Declaration of competing interest

The authors declare that they have no known competing financial interests or personal relationships that could have appeared to influence the work reported in this paper.
