# Supplementary material for: Apical longitudinal strain: A Key prognostic echocardiographic marker in patients undergoing transcatheter aortic valve implantation
Source: Int J Cardiol Heart Vasc. 2025 Nov 19;62:101844. doi: 10.1016/j.ijcha.2025.101844 (PMC12670110; doi:10.1016/j.ijcha.2025.101844)
Supplement: Supplementary Data 1 [file mmc1.docx]

**Supplemental Table 1. Baseline clinical characteristics, laboratory findings, echocardiographic findings and treatments between low and high apical LS**

|  | **High apical LS Group**  **(n=149)** | **Low apical LS Group (n=150)** | **p-value** |
| --- | --- | --- | --- |
| **Baseline characteristics** | | | |
| Age at diagnosis, years  Male sex, n (%)  Body mass index, kg/m^2^  Past medical history  Hypertension, n (%)  Diabetes mellitus, n (%)  Dyslipidemia, n (%)  Smoking, n (%)  Previous MI, n (%)  Previous PCI, n (%)  Atrial fibrillation, n (%)  STS-PROM, % (median [IQR])  Low risk (<4%), n (%)  Intermediate risk (4-8%), n (%)  High risk (≥8%), n (%) | 85.1±5.2  41(30)  22.0±3.4  108 (79)  33 (24)  77 (56)  3 (2)  3 (2)  27 (20)  5 (4)  6.3 (4-8)  28 (20)  59 (43)  36 (26) | 85.5±5.0  54 (39)  22.4±3.4  108 (78)  38 (27)  74 (53)  8 (6)  7 (5)  43 (30)  29 (20)  6.9 (4-9)  19 (14)  53 (38)  40 (29) | 0.55  0.13  0.42  0.88  0.58  0.63  0.21  0.34  0.41  <0.001  0.12  0.34  0.45  0.43 |
| **Methods of TAVI** | | | |
| Transfemoral approach  Balloon expandable valve  Valve-in-valve, n (%) | 127 (92)  98 (71)  4 (3) | 130 (93)  98 (70)  4 (3) | 0.82  0.90  1.00 |
| **Laboratory findings before TAVI procedure** | | | |
| hs-cTnT  Log-transformed hs-cTnT  BNP, pg/ml  Log-transformed BNP  eGFR, ml/min/1.73m^2^ | 0.03±0.06  -3.78±0.73  219±348  4.89±0.98  49.4±17.8 | 0.05±0.13  -3.54±0.76  348±387  5.34±1.02  47.9±15.3 | 0.20  0.01  0.002  <0.001  0.44 |
| **Echocardiographic findings before TAVI procedure** | | | |
| LAVI, ml/m^2^  IVSTd, mm  LVPWTd, mm  LVEF, %  E/e’ ratio  TRPG, mmHg  Aortic regurgitation, n (%)  Mitral regurgitation, n (%)  Tricuspid regurgitation, n (%)  LV-GLS, %  Apical LS  Mid LS  Basal LS | 55.1±17.7  12.3±1.9  11.9±1.7  64.8±5.3  18.4±6.9  27.4±8.9  6 (4)  12 (9)  13 (9)  14.8±2.2  18.5±2.8  13.1±2.8  11.7±3.4 | 57.2±19.4  12.9±2.3  12.4±2.4  57.1±10.3  19.5±7.7  27.6±8.6  11 (8)  16 (11)  14 (10)  10.5±2.6  11.0±2.9  10.3±3.1  9.2±3.1 | 0.46  0.30  0.04  <0.001  0.29  0.86  0.32  0.55  1.00  <0.001  <0.001  <0.001  <0.001 |
| **Severity of aortic stenosis** | | | |
| Trans-aortic valve velocity, m/sec  Aortic valve area, cm^2^ | 4.68±0.68  0.62±0.18 | 4.50±0.66  0.57±0.18 | 0.02  0.02 |
| **Treatments after TAVI procedure** | | | |
| RAS inhibitor, n (%)  CCB, n (%)  β-blocker, n (%)  MRA, n (%)  SGLT-2 inhibitor, n (%)  Diuretics, n (%) | 70 (51)  91 (66)  32 (23)  13 (9)  3 (2)  43 (31) | 79 (56)  68 (49)  41 (30)  41 (30)  4 (3)  68 (49) | 0.26  0.04  0.26  <0.001  1.00  0.03 |

Abbreviations: MI, myocardial infarction; TAVI, transcatheter aortic valve implantation; hs-cTnT, high sensitivity cardiac troponin T; BNP, B-type natriuretic peptide; eGFR, estimated glomerular filtration rate; LAVI, left atrial volume index; IVSTd, interventricular septal thickness in diastole; LVPWTd, left ventricular posterior wall thickness in diastole; LVEF, left ventricular ejection fraction; TRPG, transtricuspid pressure gradient; LV-GLS, left ventricular-global longitudinal strain; RAS, renin angiotensin aldosterone system; CCB, calcium channel blocker; MRA, mineralocorticoid receptor antagonist; SGLT-2, sodium glucose cotransporter 2.

The p values were obtained by student’s t-test or chi-squared test.
